# Barriers and facilitators to the implementation of social robots for older adults and people with dementia: a scoping review

**DOI:** 10.1186/s12877-021-02277-9

**Published:** 2021-06-09

**Authors:** Wei Qi Koh, Simone Anna Felding, Kübra Beliz Budak, Elaine Toomey, Dympna Casey

**Affiliations:** 1 National University of Ireland Galway, H91 E3YV Galway, Ireland; 2 German Center for Neurodegenerative Diseases Witten, Stockumer Str. 12, 58452 Witten, Germany; 3 University of Limerick, V94 T9PX Limerick, Ireland

**Keywords:** Social robots, Implementation, Barriers, Facilitators, Scoping review, Consolidated framework for implementation research, Dementia, Older people

## Abstract

**Background:**

Psychosocial issues, such as social isolation and loneliness among older adults and people with dementia, continue to pose challenges with a rapidly aging population worldwide. Social robots are a rapidly emerging field of technology, developed to help address the psychosocial needs of this population. Although studies have reported positive findings regarding their psychosocial benefits, their implementation in real-world practice remains a challenge. Nevertheless, little is known about the factors affecting their implementation. The purpose of this review is to provide a systematic overview of the barriers and facilitators affecting the implementation of social robots for older adults and people with dementia.

**Method:**

The Arksey and O’Malley approach with methodological enhancement by Levac et al. was used to guide the conduct of this review. Seven electronic databases were searched. In addition, hand searching and backward citation tracing was conducted. Three independent reviewers were involved in the screening and data charting process. Findings were synthesised and categorised into the five domains outlined in the Consolidated Framework of Implementation Research (CFIR).

**Results:**

A total of 53 studies were included in the final review. Most of the included studies were based in participants’ homes and in care facilities. Barriers and facilitators were mapped onto 18 constructs in the five domains of the CFIR. The most frequently cited barriers were mapped to the constructs within the domain of “Intervention characteristics”, where issues such as the complexity of using the technology and technical obstacles impeded implementation. Most facilitators were mapped onto the domain “Patient needs and resources”. Overall, existing research are disproportionately focused on the internal validity (i.e. characteristics) of social robots, and there is significantly less research investigating their external validity, such as organisational or wider contextual factors that can affect their implementation in real-world practice.

**Conclusion:**

This review has identified and synthesised the breadth of evidence on the barriers and facilitators to the implementation of social robots for older adults and people with dementia. Future research should pay more attention to investigating the contextual factors, using an implementation framework, to identify barriers and facilitators to guide the implementation of social robots.

**Supplementary Information:**

The online version contains supplementary material available at 10.1186/s12877-021-02277-9.

## Introduction

Populations are aging worldwide [1]. It is estimated that 5–8% of the world’s older population live with dementia [2]. Since the prevalence of dementia increases with age [3], it is one of the biggest challenges of a rapidly aging population. Previous research has identified several psychosocial challenges associated with aging and onset of dementia including social isolation, loneliness and a loss of autonomy [4, 5]. These challenges have continued to place constraints on healthcare costs and caregiving demands [6], which can influence the sustainability of care. Social robots are a rapidly emerging field of technology to facilitate social networks between people, and to interact with people in a meaningful way [7–9]. They provide a multitude of services such as affective therapy, cognitive training and companionship [10] and may be categorised into three operational groups based on their functions: (i) socially assistive robots, (ii) pet robots (or robopets), and (iii) telepresence robots. Socially assistive robots have several functions to assist users with tasks [11], pet robots are intended as viable substitutes to live animals [12] and function as pet therapy to provide physiological and emotional benefits for users [13]. Finally, telepresence robots contain a video conferencing system mounted on a mobile robotic base, and have a primary function to provide social interaction between humans [14]. As such, social robots are considered as a promising technological solution to mitigate some of the challenges associated with rapidly ageing populations by supporting psychosocial needs and assisting with care. A growing body of research focused on developing and evaluating social robots for older people and people with dementia reflects this interest. Their impact and effectiveness have been investigated and synthesized in several reviews [13, 15–17]. Although the overall evidence is not definitive due to insufficient of high-quality studies and smaller sample sizes, synthesised evidence has repeatedly demonstrated strong face validity of their positive impacts in several psychosocial domains, including reduced loneliness, improved social engagement, mood and quality of life [13, 15–17]. Despite their promise to positively impact the psychosocial health of older adults and people with dementia, their implementation in real-practice remains a challenge [18, 19]. For example, while 80% of nursing homes in Denmark have implemented Paro, a pet robot [20], only one dementia care facility has implemented Paro in Ireland [21]. For social robots, the challenges to implementation may be attributed to multi-level factors affecting implementation in actual practice, such as competing demands on the care provider [15], that may not be present or investigated in a research trial due to existence of research supported resources [22]. Additionally, the traditional stepwise approach of research (i.e. investigating implementation only after confirmatory findings of efficacy and effectiveness) has been argued to contribute to the time lag between research discovery and their uptake in real practice [23–25]. To improve the speed of knowledge creation and to improve the clinical relevance of social robots in real-world practice, it is important to pursue knowledge on the implementation of social robots alongside investigation into their effectiveness [26, 27]. Nevertheless, little is known about factors affecting their implementation in practice. A scoping review conducted by Hung et al. [15] found that infection concerns, cost and work load, stigma and ethical issues were key barriers that influenced the adoption of Paro in care settings. In another recent systematic review, Papadopoulos et al. [28] found that facilitators supporting the implementation of socially assistive robots in health and social care settings include the social robots’ usability and personalisation, users’ enjoyment and familiarity with the technology, while barriers relate to technical issues, limited capabilities of the robots, and users’ negative preconceptions. In both two reviews, an implementation framework was not used to guide the search and evidence syntheses, which highlights the possibility that some factors affecting implementation may have been overlooked. Furthermore, there is a variety of terminologies that have been used to describe implementation, which can pose challenges in evidence synthesis [29]. For instance, the term ‘implementation’ was not used in Papadopoulos et al’s [28] search strategy; instead, other terms such as ‘service evaluation’ and ‘acceptability’ were used. This issue of terminology variation has also been articulated in another review investigating determinants of implementing e-Health for caregivers of people with dementia, where authors reported that only one out of 46 included articles used the term “implementation” in the title of their publications [30]. There has been no other previous research that has provided a broad overview of the available evidence in this field. Therefore, the objectives of this review were to 1) identify the terminologies that have been used to describe implementation in relation to social robots, and 2) broadly examine existing evidence on barriers and facilitators affecting the implementation of social robots for older adults and people with dementia, and to collate and map the types of available evidence to identify potential research gaps. To address these objectives, a scoping review methodology was identified to be the most appropriate [31].

### Conceptual framework

The Consolidated Framework for Implementation Research (CFIR) was developed by Damschroder and colleagues, based on the integration of 19 different implementation theories, to enable a systematic exploration of multi-level contextual factors that can influence the implementation of an innovation or intervention [32]. There are 39 constructs across the five key domains in the CFIR that are reported to influence implementation:

1. Intervention characteristics, which refers to the key attributes of the intervention
2. Outer setting, which refers to external influences on implementation
3. Inner setting, which refers to features of the implementing organisation
4. Characteristics of individuals involved in implementation
5. Implementation process, which refers to the strategies employed in implementation

The CFIR provides a comprehensive approach to the investigation of multi-level barriers and facilitators that can influence implementation. Therefore, employing this framework will enable the identified barriers and facilitators to be presented in a structured and systematic manner. It will also allow findings to be easily compared to other implementation studies to identify research gaps.

## Methods

### Protocol and registration

The Arksey and O’Malley framework [31] for scoping reviews with methodological enhancements by Levac et al. [33], and the Preferred Reporting Items for Systematic Reviews and Meta-analysis Extension for Scoping Reviews (PRSIMA-ScR) [34] (Additional file 1) was used to guide the development, conduct and reporting of this review. The protocol was registered on the Open Science Framework (https://osf.io/2x3y9/), and the methods were described in detail in a published protocol [35].

### Stage 1: research question

The main research question governing this review was: “what is the existing evidence on the barriers and facilitators that affect the implementation of social robots for older people, including people with dementia?”

### Stage 2: identifying relevant studies

A total of seven electronic databases were searched in May 2020, and updated in November 2020: MEDLINE via Ovid, EMBASE, PsycINFO via Ovid, Scopus, Web of Science, Compendex and PubMed. A search strategy was developed in consultation with an expert research librarian using the key terms “older adults”, “people with dementia”, “social robots” and “implementation”. Various terminologies have been used across the literature to describe the concept of implementation. Therefore, we drew on an existing taxonomy of implementation outcomes by Proctor et al. [36] to define the constructs of interest and implementation search terms. They include acceptability, adoption, appropriateness, costs, feasibility, fidelity, penetration and sustainability. A full search strategy for Medline is provided in Additional file 2. We anticipated that the terms “barriers” and “facilitators” may only be discussed in the full-text of articles, potentially described using other terms. As such, these terms were excluded from the search strategy to enable a more thorough search of all research in the field. Consequently, this information was assessed through reading the full texts at a later phase of screening to ensure that no potentially relevant articles were omitted. To identify other potentially relevant studies, the reference list of reviews that were excluded from this study were manually searched [37].

### Stage 3: selection of studies

All search records were imported into Endnote and deduplicated for screening. A two-phased screening process was undertaken by three reviewers (WK, SF, KB). WK screened all articles, while SF and KB each conducted screening of 50% of all articles independently in each phase. All reviewers met to discuss the results and conflicts after each stage of screening. Firstly, titles and abstracts resulting from the search strategy were selected if they met the following inclusion criteria: (i) used a social robot for more than one session, (ii) involve older adults and/or people with dementia, (iii) contains any terms relevant to any constructs related to implementation, based on Proctor’s taxonomy, (iv) published in English language and (v) contains information about barriers and facilitators that influenced implementation. Correspondingly, the exclusion criteria were: (i) non-interventional papers, such as review articles or guidelines, (ii) did not use a social robot, or only used the social robot for a single session, (iii) did not contain any terms relating to implementation and (iv) non-English language publications. Next, full text of relevant papers were then assessed for eligibility for inclusion using the same criteria.

### Stage 4: data charting

A standardised charting form was developed using Microsoft Excel to identify key characteristics of each study, as well as barriers and facilitators to the implementation of social robots. Data that were charted included: authors, publication year, country in which the study was conducted, aims and objectives, study design, study setting, name and type social robot used, intervention characteristics, and barriers and facilitators that influenced implementation. Terms that were used to describe implementation in relation to social robots were charted from the title and abstract of studies. The charting sheet was pre-tested by all reviewers to ensure consistency in data extraction. Three reviewers were involved in data charting – WK independently charted all included articles, while SF and KB each charted 50% of the included articles. All reviewers consulted after the data charting to resolve any inconsistencies.

### Stage 5: collating, summarising, and reporting the results

WK deductively coded the extracted data by mapping determinants (i.e. barriers or facilitators) onto the 39 constructs in CFIR (Additional file 3). Coded data that were mapped onto each construct were listed, presented in a tabular form and grouped into subcategories. The synthesised results were then organised and presented categorically, based on the five domains in the CFIR. Terms used to describe implementation were mapped onto Proctor’s taxonomy of implementation outcomes, and those that are not described in the taxonomy were identified as independent terms. The frequency in which these terms were used were presented.

## Results

**Fig. 1:**
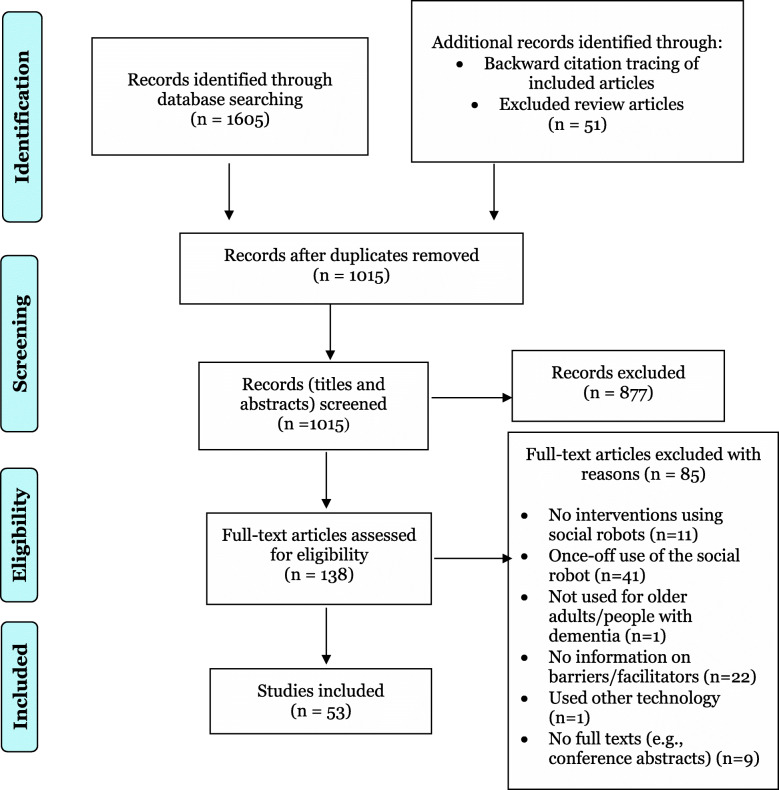
PRISMA Flow Diagram

The search of databases yielded a total of 1065 publications and an additional 51 from hand searching. After title/abstract screening, 138 articles remained for full-text screening. A total of 85 publications were excluded after full-text screening (details provided in Additional file 4), and 53 publications that met the eligibility criteria were included in the final review (PRISMA flowchart in Fig. 1). Of these, 18 were published conference papers, and 35 were journal publications.

### Study characteristics

The included publications employed three types of research methods: 15 quantitative (*n* = 15), 19 qualitative (*n* = 19) and 19 mixed-method or multi-method (*n* = 19). Studies were conducted in 19 different countries. Most were conducted within 13 countries in Europe (*n* = 37), including Austria, Belgium, Denmark, Finland, France, Greece, Germany, Hungary, Ireland, Italy, the Netherlands, Sweden, and Poland. Others were conducted in Australia (*n* = 9), the United Kingdom (*n* = 7), the United States (*n* = 5), New Zealand (*n* = 3), Japan (*n* = 2) and Mexico (*n* = 1). The majority were conducted in participants’ homes (*n* = 26) and long-term care facilities (*n* = 23). Most studies involved older adults (*n* = 31), and people with mild cognitive impairment or dementia (*n* = 24). Some studies also included other stakeholders such as care professionals or management staff (*n* = 16) and family members (*n* = 12). Table 1 shows a summary of the characteristics of included studies.

**Table 1:** Characteristics of included studies

Author	Country	Publication type	Methodology	Study design	Study participants	Study setting
Aaltonen et al., 2017 [38]	Finland	Conference paper	Qualitative	Qualitative interviews, observations	Older person, care staff, family members	Participants’ homes
Bajones et al., 2018 [39]	Austria, Greece, Sweden	Journal paper	Multi-method	Field trial	Older people (living alone, fallen in the last 2 years, and impairments in mobility,	Participants’ homes
Bajones et al., 2019 [40]	Austria, Greece, Sweden	Journal paper	Multi-method	Field trial	Older people (living alone)	Participants’ homes
Barrett et al., 2019 [41]	Ireland	Journal paper	Quantitative	Single group, pre-post pilot study	People with dementia	Nursing home
Bemelmens et al., 2016 [42]	Netherlands	Journal paper	Multi-method	Feasibility study	People with dementia, care staff, family members	Care institution for psychogeriatric care
Blond, 2019 [43]	Denmark, Finland	Journal paper	Qualitative	Ethnographic study	Older adults, care staff, management staff	Elderly care center
Bradwell et al., 2020 [44]	UK	Conference paper	Qualitative	Longitudinal study	Older people	Supported living facility
Broadbent et al., 2014 [45]	New Zealand	Conference paper	Quantitative	Repeated measures randomised cross-over trial	Older people	Participants’ homes
Caleb-Solly et al., 2018 [46]	UK, Netherlands	Journal paper	Quantitative	Usability and user experience evaluation	Older people	Assisted living studio, residential care, and participants’ homes
Carros et al., 2020 [47]	Germany	Conference paper	Qualitative	Pre and post interviews	Older people, caregivers and manager	Care home
Chang et al., 2013 [48]	USA	Conference paper	Multi-method	Observations and interview	Older people, care staff	Retirement community (long- and short-term care)
Chang et al., 2015 [49]	USA	Conference paper	Multi-method	Field study	Older people (majority had dementia), staff, visitors	Nursing home
Cruz-Sandoval et al., 2018 [50]	Mexico	Conference paper	Quantitative	Observational	Older people with dementia	Geriatric residence
de Graaf et al., 2015 [51]	UK	Journal paper	Qualitative	Exploratory in-depth study using video recording and interviews	Older people	Participants’ homes
Demange et al., 2018 [52]	France	Journal paper	Quantitative	Quasi-experimental (pre-post)	Older people with dementia	Hospital
D’Onofrio et al., 2019 [53]	Italy	Conference paper	Quantitative	Pre-post	Older people with dementia	Hospital
D’Onofrio et al., 2019 [53]	Italy, Ireland and UK	Journal paper	Quantitative	Pre-post	People with dementia	Community setting, nursing home and hospital
Fattal et al., 2020 [54]	France	Journal paper	Quantitative	Pre-post	Older people	Hospital
Fiorini et al., 2020 [55]	Italy	Conference paper	Quantitative	Pre-post	Older people	Participants’ homes
Gross et al., 2012 [56]	Netherlands Belgium	Conference paper	Qualitative	Field trial	Older people with mild cognitive impairment and their partner	Smart home (Test home)
Gross et al., 2015 [57]	Germany	Conference paper	Multi-method	Case study	Older people	Participants’ homes
Gross et al., 2019 [58]	Germany	Conference paper	Multi-method	Case study	Older people	Participants’ homes
Hebesberger et al., 2017 [59]	Austria	Journal paper	Mixed method	Concurrent multistrand research design	Older people with dementia, care staff and management staff	Hospital
Hudson et al., 2020 [60]	USA	Journal paper	Qualitative	Descriptive qualitative	Older people	Participants’ homes
Huisman and Kort, 2019 [61]	Netherlands	Journal paper	Mixed method	Evaluation study	Older adults, care staff and board members	Geriatric care facilities
Kelly et al., 2020 [62]	USA	Journal paper	Quantitative	Feasibility study	Older people with dementia	Hospital (acute care)
Khosla et al., 2017 [63]	Australia	Journal paper	Quantitative	Cross-sectional	Older people with dementia	Residential aged care facilities
Khosla et al., 2019 (Australia) [64]	Australia	Journal paper	Mixed method	Observational	People with dementia, family members	Participants’ homes
Klamer et al., 2010 [65]	UK	Conference paper	Qualitative	Case study	Older people	Participants’ homes
Kolstad et al., 2020 [66]	Japan	Journal paper	Qualitative	Semi structured interviews	Older people, nursing staff and site managers	Two nursing homes and one elderly day care centre
Kouroupetroglou et al., 2017 [67]	Italy, Ireland	Conference paper	Quantitative	Questionnaire	People with dementia	Hospital and nursing home
Melkas et al., 2020 [68]	Finland	Journal paper	Qualitative	Field study	Older people, care staff	2 care homes and a geriatric rehabilitation hospital
Moyle et al., 2013 [69]	Australia	Conference paper	Qualitative	Case study	Older people with dementia	Nursing home
Moyle et al., 2014 [70]	Australia	Journal paper	Mixed method	Semi structured interviews and observational data	Older people with dementia, care staff, family members	Long term care facilities
Moyle et al., 2016 [71]	Australia	Journal paper	Qualitative	Case study	Older people with dementia	Nursing home
Moyle et al., 2019 [72]	Australia	Journal paper	Qualitative	Descriptive qualitative	Family members of older people who live in residential care	Residential care facilities
Moyle et al., 2019 [73]	Australia	Journal paper	Qualitative	Descriptive qualitative	Older people with dementia	Long term care facility
Moyle et al., 2019 [74]	Australia	Journal paper	Qualitative	Descriptive qualitative	People with dementia, family members	Long term care facility
Niemala et al., 2017 [75]	Finland	Conference paper	Qualitative	Pre-post interviews, user observations, logged use of robot, videotaping	Older people	Long term residential home
Niemala et al., 2019 [76]	Finland	Journal paper	Multi-method	Field trial	Older people, care staff, family members	Residential care facilities
Orejana et al., 2015 [77]	New Zealand	Conference paper	Multi-method	Case study	Older people	Participants’ homes
Peri et al., 2016 [78]	New Zealand	Journal paper	Quantitative	Controlled non-randomised comparison study (Observational)	Older people, care staff, visitors	Retirement complex (Residential care ward)
Piasek and Wieczororwska-Tobis, 2018 [79]	Poland	Journal paper	Quantitative	Pre-post	Older people with mild cognitive impairment, family members	Laboratory setting and participants’ homes
Pike et al., 2020 [80]	UK	Journal paper	Qualitative	Multiple case study	Older people with dementia, family members	Participants’ homes
Portugal et al., 2019 [81]	Netherlands	Journal paper	Multi-method	Observation and post- questionnaire	Older people, care staff, visitors	Care center
Pu et al., 2020 [82]	Australia	Journal paper	Qualitative	Descriptive qualitative	Older people with dementia	Residential aged care facility
Randall et al., 2019 [83]	USA	Journal paper	Multi-method	Pre-post focus groups, survey	Older people	Participants’ homes
Sabelli et al., 2011 [84]	Japan	Conference paper	Qualitative	Ethnographic study	Older people, care staff	Elderly care center
Schroeter et al., 2013 [85]	Netherlands Belgium	Journal paper	Multi-method	Semi-structured interviews, observation, diary, questionnaire	Older people with mild cognitive impairment and their partner	Smart home (Test home)
Torta et al., 2014 [86]	Austria	Journal paper	Multi-method	Questionnaire and semi-structured interviews	Older people	Test setting (In a Senior centre)
van Maris et al., 2020 [87]	UK	Journal paper	Multi-method	Questionnaire and interviews	Older people	Retirement villages
Wu et al., 2014 [88]	France	Journal paper	Multi-method	Questionnaire and semi-structured interviews	Older people (cognitively healthy and those with mild cognitive impairment)	Test setting (In the Gerontechnology living lab in a hospital)
Zsiga et al., 2018 [89]	Hungary	Journal paper	Quantitative	Field test	Older people	Participants’ homes

### Social robots and intervention characteristics

A total of 28 different types of social robots were implemented. This includes 18 types of socially assistive robots (*n* = 33), three types of telepresence robots (*n* = 8) and five types of pet robots (*n* = 18). Paro was the most commonly deployed social robot, and was featured in 11 studies. The intervention duration ranged widely from 2 days to 4 years. Most implemented the social robot over a one-month to three-month period (*n* = 23). In terms of intervention frequency, the majority of studies (*n* = 19) implemented social robots on a full-time basis, where participants could access the social robot at any time of the day. A summary of this information can be found in Table 2.

**Table 2:** Social robot(s) and intervention characteristics

	No. of studies (n)
**Social robots used**	
*Pet robots*	*18*
Paro	11 [42, 48, 49, 52, 62, 66, 69, 72, 82, 83]
CuDDler	1 [71]
Qooboo	1 [66]
Joy for all cat	3 [44, 60, 80]
Joy for all dog	2 [44, 60]
*Telepresence robots*	*8*
VGo	1 [74]
Giraff	3 [69, 70, 74]
Double	4 [38, 55, 75, 76]
*Socially assistive robots*	*33*
Betty / Matilda	2 [63, 64]
Cafero	2 [45, 78]
CompanionAble robot	2 [56, 85]
Eva	1 [50]
Guide	1 [78]
Hobbit PT2	1 [39, 40]
iRobiQ	2 [45, 77]
Kompai mobile robot	3 [46, 88, 89]
MARIO	4 [41, 53, 67, 90]
MAX (SCITOS G3)	1 [57]
Nao / Zora	3 [61, 68, 86]
Pepper	4 [47, 54, 66, 87]
Robovie 2	1 [84]
Silbot-2	1 [43]
STRANDS robot	1 [59]
SYMPARNTER	1 [58]
Tiago	1 [79]
Violet’s Nabaztag	2 [51, 65]
**Study Duration**	
Less than 1 week	6 [53, 56, 57, 62, 81, 85]
One to four weeks	14 [39–42, 52, 54, 58, 59, 65, 67, 68, 82, 87, 88]
More than four to 12 weeks	23 [38, 45–51, 60, 64, 70–76, 78–80, 82, 86, 89]
More than 12 weeks	6 [43, 61, 63, 77, 84, 91]
No clear information	4 [53, 55, 66, 69]
**Intervention Frequency**	
Full-time (or full day)	20 [38–40, 45, 51, 55–60, 65, 77, 79, 81, 82, 85, 89]
Weekly intervention (ranging from 1 to 5 times weekly)	18 [41, 42, 47–50, 52–54, 61, 64, 67, 71–73, 75, 82–88]
Others	2 [86, 91]
No clear information	13 [43, 46, 53, 62, 63, 66, 68–70, 74, 76, 80, 84]

### Terms used to describe implementation of social robots

A total of 13 different terms have been used to describe implementation in relation to social robots (Table 3). Only 15 studies included the term “implement” or “implementation” in their title and/or abstract. Although the term “implementation” was identified in nearly half of the included studies, there appears to be a conceptual overlap on the use of this term. While some authors (*n* = 8) used this term to describe the process of using social robots within a given context [42–44, 48, 49, 68, 82, 83], others (*n* = 7) used it to describe the execution of technical or systems of the social robot [47, 56–58, 81, 85, 90]. Out of the eight constructs in Proctor’s taxonomy, we identified terms that could be mapped onto five. Overall, “acceptability” or “acceptance” were most frequently used terms (*n* = 25). Other terms that were used included use, usefulness, integration, usability and deployment.

**Table 3:** Terms used to describe implementation

Terms used	No of studies (n)
***Proctor’s taxonomy***	
acceptability, acceptance	25 [40, 41, 45, 46, 50–56, 58, 59, 62, 63, 65, 67, 77, 79, 80, 86–90]
adoption, adopt	6 [53, 60, 75, 76, 84, 88]
feasibility	8 [38, 42, 45, 54, 62, 70, 71, 74]
sustainability	1 [63]
cost	1 [72]
penetration	no data
fidelity	no data
appropriateness	no data
***Other terms***	
implementation, implement	15 [42–44, 47–49, 56–58, 68, 81–83, 85, 90]
use, usage	25 [38, 42, 44, 48, 55, 58, 60, 61, 64, 65, 68, 69, 71–73, 75, 76, 78–80, 82, 83, 85, 88, 90]
usefulness, useful	8 [45, 49, 53, 54, 63, 83, 88, 89]
integrate, integration	5 [54, 59, 60, 66, 68]
usability	4 [40, 46, 54, 55]
deploy, deployment	4 [47, 59, 81, 84]
utilisation, utilise	2 [66, 78]
employ	1 [40]

### Barriers and facilitators to implementation

A summary of barriers and facilitators coded to the CFIR, excluding constructs with no supporting data, are presented in Table 4. Overall, the barriers and facilitators were mapped onto 18 constructs across all five domains. There was no data that could be mapped onto the 21 other CFIR constructs.

**Table 4:** Summary of barriers and facilitators

CFIR construct	Barrier(s)	Facilitator(s)
**Domain 1. Innovation Characteristics**		
1.1 Relative advantage	• Relative cost as compared to other technology [70] • Less audibility [38, 75, 76]	• Sense of presence [38, 70, 74–76] • Mobility aspect [74]. • More conducive for people with dementia [70, 74] • Maintenance-free [60, 72] • Proactivity [56] • Economic advantage [59]
1.2 Adaptability	• Vocalisations [83] • Functions [45] • User interface or interaction [41, 46, 53, 63] • Physical inaccessibility [41, 47, 59, 68, 74, 77, 78, 81, 84]	• Physical accessibility [41, 74] • Customisability of interactivity or functions [47, 64]
1.3 Complexity	• Pre-programmed instructions [39, 46] • Complicated functions [39, 41, 75–77, 83, 85, 88] • Compose or program activities [61] • Multimodal interaction features [41, 67, 75, 76]	• Ease of use [39–41, 52, 57, 59–61, 65, 68, 74, 76, 81, 88]
1.4 Design quality and packaging	• Audio and speech issues [39–41, 43, 46, 47, 53, 63, 71, 74–76, 81, 84, 86, 88, 89], • Hardware problems [43, 58, 70] • Unreliable functions [39, 40, 43, 45, 46, 58, 59, 65, 71, 81, 85, 89, 90], • Unpredictable intentions [39, 40, 43, 51] • Other technical difficulties [43, 47, 54, 61, 77] • Physical attributes [16, 45, 67, 68, 72, 83] • Design [71, 72, 81, 83]	• Acceptable and/or pleasant appearance [41, 45, 54, 63, 64, 67, 68, 82, 86, 88] • Interactivity and proactivity [40, 41, 57, 58, 77, 84, 85], • Robustness [44, 57, 89]
1.5 Cost	• High acquisition and maintenance cost [44, 57, 69, 72, 77, 83, 88]	
**Domain 2: Outer setting**		
2.1 Patient needs and resources	• Unfamiliar with technology [51, 74, 88] • Cognitive impairment [41, 48, 49, 53, 67, 74, 88] • Independence in managing daily tasks [60, 77, 88] • Limited usefulness of the robot [40, 41, 45, 51, 57, 65, 83, 87] • Doubts about sustained benefits [57, 86, 88]. • Intrusiveness or privacy [45, 46, 51, 57, 83, 88] • Negative affect [40, 47, 53, 59, 64, 65, 71, 88] • Negative perceptions or stigma [40, 44, 51, 52, 54, 55, 62, 71, 80, 81, 88]	• Support and familiarisation [47, 57, 79, 88] • Emotional support [41, 52, 57, 58, 60, 82–85] • Companionship [44, 45, 60, 77, 82, 83] • Improvement to daily life [40, 58, 63, 81] • Entertainment [41, 45, 50, 63, 64] • Reminiscence [41, 45, 71] • Reminders [54, 58, 64] • Phased introduction and training [46] • Prolonged use [46, 47, 51, 70].
2.2 External policy/incentives	• Align care work with national care policy [75, 76]	
**Domain 3: Inner Setting**		
3.1 Compatibility	• Institutional regulations: privacy, space and safety privacy [38, 75, 84] • Confused/frightened residents [59] • Background noises [41, 53, 75] • Concern about misuse of technology [38, 75, 76] • Lack of support from co-workers [61] • Delineate professional boundary [38, 75, 76] • Ethical concerns [42, 68, 71, 73] • Hygiene [42, 44, 72, 73] • Interfere with routine • Physical environment [40]	• Supported work of care professionals [47, 59, 68, 76, 84] • Integration into care routine [42, 47, 49, 75, 84] • Positioning of social robots [51, 60, 65] • Adaptation of physical environment [40, 41]
3.2 Relative priority	• Existing care work/processes took precedence [66, 68, 75] • Workplace tension [68]	
3.3 Leadership engagement		• Leadership involvement and commitment [61]
3.4 Available resources	• Poor network connectivity [38, 39, 55, 61, 68, 70, 74–76, 81] • Lack of manpower, time or training [42, 66, 68–70] • Computer incompatibility [74]	• Improved network infrastructure [61] • Time and support for care professionals [61].
3.5 Access to knowledge and information	• Access to support in rural areas [77]	• Dedicated helpdesk within care facility [61] • Individualised intervention instructions/manual [42, 43, 61]
**Domain 4: Characteristics of Individuals**		
4.1 Knowledge and beliefs	• Initial ambivalence/negative attitudes [42, 47, 59, 66, 68, 72, 74, 81] • Fear of damaging robot [59, 77] • Privacy concern [38, 75, 76] • Fear of job replacement [47, 59] • Negative perceptions, which stemmed from technical challenges/ perceived lack of usefulness [59, 61, 74, 75]	• Evolved attitude after witnessing positive impacts on older adults/people with dementia [42, 44, 47, 49, 56, 66, 68–70, 72, 74, 75, 80, 81] • Understanding that robots cannot replace their jobs [47] • Motivation to support robot interactions [42, 61, 84] • Alignment to organisation visions [61]
4.2 Self-efficacy	• Unequipped to program and compose activities [61]	• Gain experience over time [61]
**Domain 5: Implementation Process**		
5.1 Planning	• Assign robot with a clearly indicated role [84]	
5.2 Engaging		• Public exposure facilitated engagement and change in perceptions [49, 59, 70]
5.3 Key stakeholders	• Negative attitudes of care professionals [69]	• Care professionals’ enthusiasm [66] • Active engagement with care professionals [84] • Mediation of robot interactions [43, 47–50, 67]
5.4 External change agents	• Lack of sustainability [47]	• Support robot interactions [40, 41, 49, 74, 76] • Provide technical support [39, 43, 77]

### Domain 1: innovation characteristics

#### Relative advantage

Telepresence robots were considered to be more disadvantageous than using the telephone or skype as they were more expensive [70] and had less audibility to cater to those with a hearing impairment [38, 75, 76]. Relative advantages included an increased sense of presence due to their video element [38, 70, 74–76] and mobility aspect [74]. They were also reported to be more conducive for use with people with dementia [70, 74]. Pet robots were compared to live animals, where their maintenance-free nature was seen as an advantage [60, 72]. Socially assistive robots were perceived to be more beneficial than a tablet solution due to their proactivity [56], and potential economic profitability as compared to having human staff [59].

#### Adaptability

The inability to adapt the functions of social robots to cater to participants’ preferences and abilities impeded their use. This included the inability to adjust vocalisations [83], personalise functions [45], and customise user interfaces or modes of robot interaction [41, 46, 53, 63]. Other barriers relate to issues of physical inaccessibility [41, 47, 59, 68, 74, 77, 78, 81, 84]. Correspondingly, facilitators included the physical accessibility [41, 74] and customisability of the robots’ interactivity or functions [47, 64].

#### Complexity

The complexity of operating social robots primarily related to the use of socially assistive robots, which included complicated pre-programmed instructions [39, 46] and functions [39, 41, 75–77, 83, 85, 88], or difficulty composing or programming activities [61]. For telepresence robots, navigation difficulties occurred during remote driving [69, 75, 76]. For some participants, particularly people with dementia, the multiple modes of visual, auditory and tactile interaction with social robots were confusing and challenging [41, 67, 75, 76]. Facilitators relating to their ease of use were reported in 14 studies [39–41, 52, 57, 59–61, 65, 68, 74, 76, 81, 88], of which some attributed this to the involvement of users in the design process [41] and prolonged technology use [86].

#### Design quality and packaging

Technical issues were widely reported as barriers, particularly in relation to socially assistive robots. These included audio and speech issues [39–41, 43, 46, 47, 53, 63, 71, 74–76, 81, 84, 86, 88, 89], hardware problems [43, 58, 70], overheating [40, 69, 70], unreliability of functions [39, 40, 43, 45, 46, 58, 59, 65, 71, 81, 85, 89, 90], unclear or unpredictable actions [39, 40, 43, 51] and other technical issues [43, 47, 54, 61, 77]. The frequent need to recharge batteries was also cited as a barrier [83]. Next, barriers relating to their physical attributes, such as weight [16, 72], size [45, 68] unpleasant vocalisations [16, 67, 83] and unsatisfactory levels of interactivity [16, 83], were raised. Finally, unfamiliar designs [72, 83] and the “machine-like” [71, 81] or “toy-like” [71] appearances of social robots were also cited as issues. Facilitators were related to overall acceptable or pleasant appearances and design [41, 45, 48, 49, 54, 63, 64, 67, 68, 82, 86, 88]. Other facilitators included the interactivity and proactivity of social robots [40, 41, 57, 58, 77, 84, 85], and their overall robustness [44, 57, 89].

#### Cost

Multiple stakeholders raised concerns about high acquisition costs [44, 57, 69, 72, 83, 88], and maintenance costs of social robots, especially when used in rural areas or out of their country of manufacture [69, 77].

### Domain 2: outer setting

#### Patient needs and resources

The demographics of participants influenced their needs. Older people who were less familiar with technology were more hesitant to use social robots [51, 74, 88]. People with dementia, especially those with more cognitive impairment, required more ongoing support [41, 48, 49, 53, 67, 74, 88]. Correspondingly, familiarisation and support to use the technology was perceived to be a necessary facilitator [47, 57, 79, 88]. Next, the inability of social robots to meet participants’ needs also impeded their use. Older adults who were living at home and were independent in managing daily tasks felt that the technology was unnecessary [60, 77, 88], had limited usefulness [40, 41, 45, 51, 57, 65, 83, 87], and had doubts about their benefits with sustained use [57, 86, 88]. Issues that were raised by both older adults and people with dementia include privacy concerns [45, 46, 51, 57, 83, 88], negative affect which stemmed from technical issues [40, 47, 53, 59, 64, 65, 71, 88], and negative perceptions or stigma [40, 44, 51, 52, 54, 55, 62, 71, 80, 81, 88]. Correspondingly, when functions of the robots aligned with participants’ needs and were perceived to be relevant, their use was facilitated. The needs that these robots fulfilled included emotional support [41, 52, 57, 58, 60, 82–85], companionship [44, 45, 60, 77, 82, 83], perceived improvements to daily life [40, 58, 63, 81], entertainment [41, 45, 50, 63, 64], reminiscence [41, 45, 71] and non-intrusive reminders [54, 58, 64]. Phased introduction and training [46] and familiarisation also facilitated a greater acceptance of [70] and adaptation to the technology [46, 47, 51].

#### External policy and incentive

Only two studies (*n* = 2) reported on external policy as a facilitator, where care professionals perceived that use of the technology aligned their care work with the wider national care policy [75, 76].

### Domain 3: inner setting

#### Compatibility

In care facilities, barriers included institutional regulations which limited the mobility of social robots due to issues of privacy [38, 75], safety and space allocation [84]. The unexpected appearances of the robot confused some residents [59], and background noises also influenced participants’ interaction with the technology [41, 53, 75]. Next, challenges integrating social robots into work process included concerns about potential misuse of the technology [38, 75, 76], lack of support from co-workers [61], uncertainty on how to delineate a professional boundary [38, 75, 76], ethical [42, 68, 71, 73], and hygiene concerns [42, 44, 72, 73]. Correspondingly, they were compatible with work processes when their use supported the work of care workers [47, 59, 68, 76, 84], could be integrated into daily care routine [42, 47, 49, 75, 84]. For studies conducted in participants’ homes, incompatibility occurred when social robots interfered with daily routine [47, 51], or when environment inaccessibility impeded the robots’ mobility [40, 51, 58]. Facilitators included an integrated routine of use [51, 60, 65], and environment accessibility [40, 41].

#### Relative priority

Barriers relating to relative priority were reported in three studies (*n* = 3), where care professionals felt that social robots caused additional work, and that existing work took precedence [66, 68, 75]. Their use also led to workplace tension, where those who did not prioritise use of the technology dissented those who used it [68].

#### Leadership engagement

Only one study (*n* = 1) reported on leadership engagement as a facilitator, where organisational leaders demonstrated active involvement and commitment towards implementation effort. Support services and meetings were planned for care professionals to exchange knowledge and experiences [61].

#### Available resources

More resource-related barriers than facilitators were identified. In care facilities, barriers included poor network connectivity [38, 39, 61, 68, 70, 74–76, 81], and lack of manpower, time or training [42, 66, 68–70]. Only one study reported on facilitators, where the network infrastructure was boosted, and time and support were provided to support use of the technology [61]. For studies that were conducted in participants’ homes, or involved family members who lived at home, resource barriers include a lack of Wi-Fi infrastructure [55] and computer incompatibility [74] to connect with the robot at the care facility.

#### Access to knowledge and information

Access to technical support was reported as a barrier for participants who lived in rural areas [77]. Three studies reported access to knowledge and information within care facilities through a dedicated helpdesk [61], a manual and individualised interventions instructions [42, 43], which supported implementation.

### Domain 4: characteristics of individuals

#### Knowledge and beliefs

Some care workers and family members were ambivalent or had negative attitudes towards social robots [42, 47, 59, 66, 68, 72, 74, 81], hesitated their use for fear of damaging them [59, 77], and had concerns about privacy [38, 75, 76] and job replacement by robots [47, 59]. While some negative perceptions persisted after experiencing their use, due to technical challenges or perceived lack of usefulness [59, 61, 74, 75], other attitudes evolved positively after witnessing their positive impacts [42, 44, 47, 49, 56, 66, 68–70, 72, 74, 75, 80, 81], and having a renewed understanding that robots cannot replace their jobs [47]. As such, they were motivated and willing to support robot interactions [42, 61, 84]. Perceptions at the managerial level were only reported in one study (*n* = 1), which reported positive views that the technology aligned with the organisation’s vision [61].

#### Self efficacy

Only one study (*n* = 1) reported that care workers felt unequipped to compose group activities using social robots. Nevertheless, they gained experience to work around the capabilities of the technology over time [61].

#### Planning

In one study (*n* = 1), the plan to assign a social robot with a clear role to make it more approachable facilitated the implementation process [84].

#### Engaging

The public exposure of social robots facilitated engagement by multiple stakeholders [59], who developed positive perceptions of the value of the technology from observing robot interactions [49, 70].

#### Key stakeholders

Negative attitudes of care professionals was reported as a key barrier to implementation [69], while staff enthusiasm was facilitated their use [66]. Only one study (*n* = 1) reported active involvement of care professionals in the implementation process, which facilitated their proactivity and enthusiasm [84]. Staff-mediated robot interactions, such as using active strategies to mediate the limitations of robot interactions [43, 47, 49, 50, 67] and changing composition of group sessions [48] led to more successful robot interactions.

#### External change agents

Eight studies (*n* = 8) identified family members, researchers and robot developers to be external change agents, who facilitated the implementation process by supporting participants’ interactions with social robots [40, 41, 49, 74, 76] and providing technical support [39, 43, 77]. However, the ethical challenge of lack of sustainability of social robot intervention after the end of the study was reported in one study [47].

## Discussion

This review synthesises available evidence on the barriers and facilitators to the implementation of social robots for older people and people with dementia. Most included studies were conducted in long term care facilities and in participants’ homes, and the majority used socially assistive robots and pet robots. The most frequently cited barriers were mapped onto constructs within the domain “Intervention characteristics”, while most facilitators were mapped onto the domain “Patients needs and resources”.

### Terminology

Overall, less than a third of the articles included the term “implementation” in their title and/or abstracts. There appears to be no clear conceptual definition of the term “implementation”. This could be attributed to different disciplinary research focus and/or discipline-specific vocabulary, since included papers were derived from different academic fields: health and social sciences, engineering and computer science. In health and social science contexts, implementation refers to “the constellation of processes intended to get an intervention into use within an organisation” [32]. However, in computer science, it is used to describe the process of executing technical applications [92]. Given that social robotics is a transdisciplinary field, it is important for researchers to be aware of discipline-specific terms. Moving forward, a concept analysis should be done to understand interdisciplinary concepts used to describe implementation in relation to social robots. Terms in Proctor’s taxonomy were identified in titles and/or abstracts of most included papers. This highlights the practicability of using the taxonomy to develop a sensitive search strategy to identify studies that investigated intervention implementation.

### Barriers

Barriers to implementation were primarily related to the characteristics of social robots (i.e. “Intervention characteristics” domain), such as complexity, physical accessibility and cost. In particular, technical failures were raised as issues in more than half of the included studies. It may be worth noting that most of these barriers were related to the use of socially assistive robots. This may be attributed to the range of functions available on such robots (as compared to telepresence or pet robots), which can proportionately increase the complexity of their operation. Although another possible explanation for barriers in this domain are that many of the social robots that were used were prototypes, it is also important to note that such issues were also raised in relation to the use of commercially available social robots such as Zora, Pepper and Giraff. Such challenges are not novel to social robots, as similar issues have been well-documented even amongst studies which used less novel or daily technology to conduct interventions [30, 93–95]. These issues had repercussions on other implementation domains, as they resulted in negative perceptions by multi-level key stakeholders, including older people and people with dementia, family members and care professionals. This finding is in alignment with findings by Rozental et al. [96], which found that such technical problems evoked negative psychological effect among users.

People with cognitive impairment required more support to use social robots, and those with less experience with technology had lower self-efficacy. This finding corresponded with existing research [15, 16, 97, 98]. Next, the mismatch between the social robots’ function and users’ needs was also reported as an obstacle. Such barriers were primarily reported in studies which investigated the use of social robots for cognitively older adults who were living at home, suggesting that their needs and expectations of social robots differ from people with dementia or are living in care facilities, who may use technology differently. A recent scoping review by Abdi and colleagues [99] found that the needs of community-dwelling older adults ranged widely from mobility needs and interpersonal needs to self-management needs. As such, they may require social robots to have more functionalities that are tailored to their needs [83, 100]. In contrast, the needs of people with dementia and those in care setting differed. They included having stimulating day time activities and company [101]. Understanding of the needs of intended population is a therefore fundamental contextual consideration for implementing social robots.

Although one of the key bases for the development of social robots is to support and aid caregiving in individuals’ homes and care settings [102], which is expected to be increasingly strained due to a rapidly aging population [15, 103], there is ironically a lack of studies which has investigated how social robots can be successfully integrated into care organisations (i.e. “Inner setting” domain). There were significantly more barriers than facilitators identified in this CFIR domain. These barriers, including incompatibility of the intervention to institutional regulations or work processes and the lack of time, manpower and training to support implementation efforts, corresponding with existing literature [104, 105]. Therefore, dedicated resources should be allocated to supported the implementation of social robots, especially during the initial implementation phase [106] to allow care organisations and care professionals to familiarise and adapt to their use [107]. Next, even though organisational theories have highlighted the influence of other external factors on implementation such as external policies or incentives [108, 109], this was only reported in two studies. There is also a lack of studies that reported perspectives of other stakeholders, such as management staff and policy makers, which highlights research gaps in these areas. Finally, findings relating to the CFIR domain of “Implementation process” were scarce as there were few studies that undertook process evaluations.

### Facilitators

Most of the identified facilitators correspond with the identified barriers. For instance, the characteristics of the social robots, such as their physical accessibility, ease of use, cost and technical robustness were identified as implementation facilitators. In addition, the match between social robots’ functions and users’ needs and their compatibility with work processes within care organisations were seen as enablers. We also found that despite initial ambivalence or scepticism, older adults and people with dementia developed positive perceptions after using social robots with functions that matches their needs or expectations. Similarly, when family members and care professionals experienced the positive impacts of the technology and developed a renewed understanding that they cannot replace their jobs, positive attitudes were reported. This confirms current research findings that direct experiences with a technology can elicit attitude change when the interactions evoke cognitive-affective discrepancies from baseline beliefs [110, 111]. These positive perceptions had implications on other implementation domains. In the CFIR domain of “Implementation process”, care professionals and family members who had had positive attitudes were more enthusiastic in supporting and facilitating robot interactions. The mediation of robot interactions by these stakeholders also helped to reconcile the limitations of the intervention characteristics, such as technical issues and the complexity of use. These facilitators also highlight the importance of avoiding evaluating implementation determinants in silos, and instead consider the interplay of multi-level contextual factors that influence implementation [112, 113].

### Future research and practical implications

Overall, more barriers than facilitators were identified. Data from this review could only be mapped onto 18 out of 39 constructs in the CFIR. Data were mostly coded to the CFIR domain of “intervention characteristics”, and there is significantly less research emphasis on other CFIR domains. This is also exemplified through the lack of data that could be mapped onto 21 other CFIR constructs. This indicates that existing research have been focused on the internal validity of the intervention, and that future research focus must be directed towards identifying other contextual factors that can influence the external validity of social robots in real-world practice. Very few of the included studies have undertaken process evaluations, and none have used an implementation framework to ensure a systematic approach to consider all factors that can affect implementation. Given the complexity of implementing social robots, process evaluations can provide valuable insights that may explain why the intervention has (or has not) been implemented as intended in real-world practice [114], and how different contextual factors may have influenced overall intervention outcomes [115]. Future research should also consider applying an appropriate theoretical framework to guide a thorough investigation of implementation determinants, which can then enable corresponding strategies to be identified and tested in real-world practice. Waltz and colleagues [116] developed a tool for mapping barriers identified on each CFIR domain to the Expert Recommendations for Implementing Change (ERIC), which contains a comprehensive collection of implementation strategies [117]. For instance, to address a barrier relating to “compatibility”, one recommended strategy listed in CFIR-ERIC mapping tool is to conduct local consensus discussions, where different key stakeholders should engage in active discussions about whether social robots are appropriate to address needs within their context. Finally, aside from focusing on barriers, it is also pivotal to leverage on facilitators to guide the successful implementation of social robots in the real-world setting.

## Strengths and limitations

There are a number of strengths underpinning this work. First, the methodological framework that was used was transparent and rigorous. We searched multiple databases, including grey literature and engineering databases. The application of an implementation science framework (i.e., the CFIR) enabled results to be presented in a comprehensive and systematic manner. Nevertheless, there are limitations of this review. In our review protocol, we reported a plan to extract terms used to describe implementation from the full text of included articles. However, due to the large number of articles that were included in this review, we had to deviate from the protocol to only chart terms that were included in the title and/or abstract of included papers. Articles that were published in other languages were not included in this review. Hence, relevant studies might be missed. In addition, the review aggregated barriers and facilitators related to the implementation of social robots in participants’ home and long-term care settings, and thus the findings mainly apply to these settings. Several different social robots (i.e., interventions) were included in this review. The heterogeneity of the interventions and study settings could be a fundamental limitation, as these variable factors can affect implementation differently. Nevertheless, implementation barriers and facilitators that were identified in this study revolved around similar themes.

## Conclusion

This review has identified and synthesised terms used to describe implementation in relation to social robots, and the breadth of evidence on the barriers and facilitators to the implementation of social robots for older adults and people with dementia. There is a lack of clear conceptual clarity regarding the term “implementation”. A concept analysis may be warranted to explore this topic in depth. Although social robots show promise for improving the psychosocial health of older adults and people with dementia, there has been little attention paid to their implementation in the real-world setting. Most existing research were focused on evaluating the characteristics of social robots, and there has been significantly less research which investigated other multi-level contextual factors, such as organisational or wider contextual factors, that can influence their implementation in real-world practice. Further research in these domains, using an implementation framework, is necessitated.

## Supplementary Information

**Additional file 1.**

**Additional file 2.**

**Additional file 3.**

**Additional file 4.**

## Data Availability

Supporting data and materials used in this paper can be accessed online through various public databases. The datasets used and/or analysed during the current study are available from the corresponding author on reasonable request.

## Abbreviation

CFIR: Consolidated Framework of Implementation Research
