# Supplementary material for: Barriers and facilitators to the implementation of social robots for older adults and people with dementia: a scoping review
Source: BMC Geriatr. 2021 Jun 9;21:351. doi: 10.1186/s12877-021-02277-9 (PMC8191065; doi:10.1186/s12877-021-02277-9)
Supplement: Supplementary file 2 — Additional file 2. [file 12877_2021_2277_MOESM2_ESM.docx]

**Medline Search Strategy**

1. (social* and robot*).mp. [mp=title, abstract, original title, name of substance word, subject heading word, floating sub-heading word, keyword heading word, organism supplementary concept word, protocol supplementary concept word, rare disease supplementary concept word, unique identifier, synonyms]

2. Aged/

3. (age or elderly or senior citizen* or older adult).mp. [mp=title, abstract, original title, name of substance word, subject heading word, floating sub-heading word, keyword heading word, organism supplementary concept word, protocol supplementary concept word, rare disease supplementary concept word, unique identifier, synonyms]

4. dementia.mp. or Dementia/

5. implement*.mp.

6. quality improvement.mp. or Quality Improvement/

7. program evaluation.mp. or Program Evaluation/

8. dissemination.mp. or Information Dissemination/

9. "Patient Acceptance of Health Care"/ or acceptability.mp.

10. satisfaction.mp. or Personal Satisfaction/

11. adoption.mp.

12. uptake.mp.

13. "Delivery of Health Care"/ or utili*ation.mp.

14. appropriateness.mp.

15. cost.mp. or "Costs and Cost Analysis"/

16. Feasibility Studies/ or feasib*.mp.

17. fidelity.mp.

18. sustainability.mp

19. penetration.mp.

20. 2 or 3 or 4

21. 5 or 6 or 7 or 8 or 9 or 10 or 11 or 12 or 13 or 14 or 15 or 16 or 17 or 18 or 19

22. 1 and 20 and 21
