# Supplementary material for: Barriers and facilitators to the implementation of social robots for older adults and people with dementia: a scoping review
Source: BMC Geriatr. 2021 Jun 9;21:351. doi: 10.1186/s12877-021-02277-9 (PMC8191065; doi:10.1186/s12877-021-02277-9)
Supplement: Supplementary file 3 — Additional file 3. [file 12877_2021_2277_MOESM3_ESM.docx]

**CFIR Codebook and Coding Guidelines**

The codebook below is the result of the operationalisation/adaptation of existing definitions and eligibility criteria for CFIR constructs (available at https://cfirguide.org/) to the topic area (i.e. using social robots for older adults and/or people with dementia). The existing construct definitions and eligibility criteria are adapted to this topic area to ensure coding consistency.

1. **Intervention Characteristics**

| **CFIR Constructs** | **Description** | **Inclusion and/or Exclusion Criteria** |
| --- | --- | --- |
| Intervention source | Perception of key stakeholders about whether the innovation is externally or internally developed. | Inclusion Criteria: Include statements about the source of the innovation and the extent to which interviewees view the social robot as internal or external to their organization or setting  Exclusion Criteria: Exclude statements related to who participated in the decision-making process to implement social robots and code to **“Engaging”**, as an indication of early (or late) engagement. |
| Evidence strength & quality | Stakeholders’ perceptions of the quality and validity of evidence supporting the belief that social robots will have desired outcomes | Inclusion Criteria: Include statements regarding awareness of evidence and the strength and quality of evidence, as well as the absence of evidence or a desire for different types of evidence instead of evidence from the literature.  Exclusion Criteria: Exclude or double code statements regarding the receipt of evidence as an engagement strategy to Engaging: Key Stakeholders. |
| Relative Advantage | Stakeholders’ perception of the advantage of implementing social robots compared to the status quo or an alternative. | Inclusion Criteria: Include statements about stakeholders’ perceptions that the social robot is better (or worse) than the status quo or an alternative intervention.  Exclusion Criteria: Exclude statements that do or do not demonstrate a strong need for social robot and/or that the current situation is untenable (e.g. statements that social robots are absolutely necessary or absolutely redundant) and code to **“Tension for change’’**. Exclude statements regarding specific needs of end users (i.e. older adults and/or people with dementia) that demonstrate a need for social robots and code to **“Users’ Needs & Resources”** |
| Adaptability | Perceived inherent ability/allowance to adapt the social robots to meet local needs | Inclusion Criteria: Include statements related to perceptions of the (in)ability to adapt the social robot.  Exclusion Criteria: Exclude statements that the social robots did or did not need to be adapted, and code to **“Compatibility”** |
| Trialability | Perceived inherent ability/allowance to trial the social robot on a small scale, and to be able to reverse course (undo implementation) if warranted. | Inclusion Criteria: Include statements related to perceptions of the (in)ability to trial (i.e. test/pilot) the social robot.  Exclusion Criteria: Exclude statements regarding results of actual or suggested trials of the social robot and code to **“Evidence Strength and Quality”** |
| Complexity | Perceived difficulty of using the social robot, reflected by duration, scope, radicalness, disruptiveness, centrality, and intricacy and number of steps required to implement. | Inclusion Criteria: Code statements regarding the complexity of the social robot (i.e. not dependent on context)  Exclusion Criteria: Exclude statements regarding the complexity of implementation and code to the appropriate CFIR code (e.g. complexity relating so space, code to **“Available Resource”,** and difficulties related to engaging users, code to **“Engaging”)** |
| Design Quality & Packaging | Perceived excellence in how the social robot is bundled, presented, and assembled. | Inclusion Criteria: Include statements regarding the quality, presentation and packaging of the social robot. Packaging relates to how the social robot is presented and even how accessible it is for end users (i.e. older adults and/or people with dementia)  Exclusion Criteria: Exclude statements regarding the presence or absence of materials and code to **“Available Resources”.** Exclude statements regarding the receipt of materials as an engagement strategy and code to **“Engaging”.** |
| Cost | Costs of the intervention and costs associated with implementing the intervention. | Inclusion Criteria: Include statements related to the cost of the social robot and its implementation.  Exclusion Criteria: Exclude statements which refer to the presence or absence of resources and code to **“Available Resources”** (e.g. money, time). |

1. **Outer Setting**

| **CFIR Constructs** | **Description** | **Inclusion and/or Exclusion Criteria** |
| --- | --- | --- |
| Needs and Resources (of end users) | The extent to which the needs of end users (i.e. older adults and/or people with dementia), as well as barriers and facilitators to meet those needs, are accurately known and prioritized by the organisation/setting | Inclusion Criteria: Include statements demonstrating (lack of) awareness of the needs and resources of end users (i.e. older adults and/or people with dementia). For example, users’ demand for social robot, barriers and facilitators experienced by end users to using social robots, end users’ satisfaction with social robot)  Exclusion Criteria: Exclude consumer feedback on whether the social robot is having the desired outcome and code to **“Evidence Strength & Quality”.** |
| Cosmopolitanism | The degree to which the care setting or organisation is networked with other external organizations (i.e. external people and groups). | Inclusion Criteria: Include descriptions of outside group memberships and networking done outside the care setting/organisation  Exclusion Criteria: Exclude statements of networking with external organisations, general networking, communication that did not exist prior to the social robot implementation and code to **“Network and Communication”** |
| Peer Pressure | Mimetic or competitive pressure to implement social robot(s); typically, because most or other key peer or competing organisations/facilities have already implemented or are in a bid for a competitive edge. | Inclusion Criteria: Include statements about perceived pressure or motivation from other entities or organisation in the local geographic area to implement social robot |
| External Policy & Incentive | A broad construct that includes external strategies to spread innovations, including policy and regulations (governmental or other central entity), external mandates, recommendations and guidelines and public reporting. | Inclusion Criteria: Include descriptions of external strategies (outside the care setting/organisation) to social robot(s) (e.g. policies, regulations, guidelines). |

1. **Inner Setting**

| **CFIR Constructs** | **Description** | **Inclusion and/or Exclusion Criteria** |
| --- | --- | --- |
| Structural Characteristics | The social architecture, age, maturity, and size of the setting | Inclusion Criteria: Include statements about the size, social architecture, age, maturity and size of the implementing organisation/setting. |
| Networks and Communications | The nature and quality of webs of social networks and the nature and quality of formal and informal communications within the care facility/implementing setting | Inclusion Criteria: Include statements about general networking, communication, and relationships in the organisation/setting, and statements related to team formation, quality, and functioning |
| Culture | Norms, values, and basic assumptions of the setting | Inclusion Criteria: Include statements related to concepts captured in the Competing Values Framework approach - four archetypical organizational cultures: team culture, hierarchical culture, entrepreneurial culture and rational culture.  (Note: Culture is often viewed as relatively stable, socially constructed, and subconscious) |
| Implementation climate | The absorptive capacity for change, shared receptivity of involved individuals to the social robot(s), and the extent to which use of that intervention will be rewarded, supported, and expected within their setting | Inclusion Criteria: Include statements regarding the general level of receptivity to implementing the social robot |
| (i) Tension for change | The degree to which stakeholders perceive the current situation as intolerable or needing change. | Inclusion Criteria: Include statements that (do not) demonstrate a strong need for the social robot and/or that the current situation is untenable (e.g. statements that the social robot is absolutely necessary or that it is redundant with other programs).  Exclusion Criteria: Exclude statement regarding specific needs of end users that demonstrate a need for the social robot, but do not necessarily represent a strong need or an untenable status quo and coe to **“Needs and Resources”**. Exclude statements that demonstrate the intervention is better (or worse) than existing programs and code to **“Relative Advantage”** |
| (ii) Compatibility | The degree of tangible fit between meaning and values attached to the social robot by involved individuals, how those align with individuals’ own norms, values, and perceived risks and needs, and how the social robot fits with existing workflows and systems. | Inclusion Criteria: Include statements that demonstrate the level of compatibility the social robot has with values and work processes of the implementing organisation/setting.  Exclusion Criteria: Exclude statements regarding the priority of the social robot based on compatibility with organizational values and code to **“Relative Priority”,** e.g., if the social robot is not prioritized because it is not compatible with organizational values. |
| (iii) Relative priority | Individuals’ shared perception of the importance of the social robot in the setting | Inclusion Criteria: Include statements that reflect the relative priority of the social robot (e.g. statements related to change fatigue in the care setting/organisation due to implementation of many other programs). |
| (iv) Organisational incentives & rewards | Extrinsic incentives such as goal-sharing, awards, performance reviews, promotions, and raises in salary, and less tangible incentives such as increased stature or respect. | Inclusion Criteria: Include statements related to whether incentive systems are in place to foster (or hinder) implementation, e.g. rewards or disincentives for staff engaging in the implementation of the social robot |
| (v) Goals & feedback | The degree to which goals are clearly communicated, acted upon, and fed back to staff, and alignment of that feedback with goals. | Inclusion Criteria: Include statements related to the (lack of) alignment of social robot implementation with larger goals of the care setting/organisation, as well as feedback to staff regarding those goals. Goals can be related to (un)stated organisational needs. e.g., regular audit and feedback showing any gaps between the current organizational status and the goal. Goals and Feedback include organizational processes and supporting structures independent of the implementation process. |
| (vi) Learning climate | A climate in which: a) leaders express their own fallibility and need for team members’ assistance and input; b) team members feel that they are essential, valued, and knowledgeable partners in the change process; c) individuals feel psychologically safe to try new methods; and d) there is sufficient time and space for reflective thinking and evaluation. | Inclusion Criteria: Include statements that support (or refute) the degree to which key components of the implementing organisation/care setting exhibit a ‘learning climate’. |
| Readiness for Implementation | Tangible and immediate indicators of the care setting/organisation to its decision to implement the social robot | Inclusion Criteria: Include statements regarding the general level of readiness for implementation.  Exclusion Criteria: Exclude statements regarding the general level of readiness for implementation that are captured in the sub-codes below. |
| (i) Leadership engagement | Commitment, involvement, and accountability of leaders and managers with the implementation. | Inclusion Criteria: Include statements regarding the level of commitment/support of leadership towards the implementation of the social robot  Double code statements regarding leadership engagement to Engaging: **“Formally Appointed Internal Implementation Leaders”** or **“Champions”** if an organizational leader is also an implementation leader, e.g., if a director of primary care takes the lead in implementing a new treatment guideline. Note that a key characteristic of this Implementation Leader/Champion is that s/he is also an Organizational Leader. |
| (ii) Available resources | The level of resources dedicated for implementation and on-going operations, including money, training, education, physical space, and time. | Inclusion Criteria: Include statements related to the presence or absence of resources described above or resources specific to implementation of the social robot  Exclusion Criteria: Exclude statements related to training and education and code to the CFIR sub-construct **“Access to Knowledge and Information”.** Exclude statements related to the quality of materials and code to the CFIR construct **“Design Quality and Packaging”.** |
| (iii) Access to knowledge & information | Ease of access to digestible information and knowledge about the social robot and how to incorporate it into work tasks. | Note: Information and knowledge includes all sources such as experts, other experienced staff, training, documentation, and computerized information systems.  Inclusion Criteria: Include statements related to stakeholder access to knowledge and information regarding the social robot. Include knowledge and information available from within the implementing care setting/organisation (e.g. training provided by staff) or those available from external entities/stakeholders to the care setting/organisation (e.g. via a health agency or supplier providing information).  Exclusion Criteria: Exclude statement related to engagement strategies and outcomes (e.g. how key stakeholders become engaged), and code to **“Key Stakeholders”.** Exclude statements about general networking, communication, and relationships in the organisation, which are independent of the social robot intervention and code to **“Networks and Communication”’**. |

1. **Characteristics of Individuals**

| **CFIR Constructs** | **Description** | **Inclusion and/or Exclusion Criteria** |
| --- | --- | --- |
| Knowledge & beliefs about the social robot | Individuals’ attitudes toward and value placed on the social robot as well as familiarity with facts, truths, and principles related to the innovation | Inclusion Criteria: Include statements related to individuals’ attitudes towards and value placed on the social robot as well as familiarity with facts, truths, and principles related to the innovation  Exclusion Criteria: Exclude statements related to familiarity with the evidence regarding social robot(s) and code to **“Evidence Strength and Quality”** |
| Self-efficacy | Individual belief in their own capabilities (confidence in their ability) to execute courses of action to achieve implementation goals (i.e. to carry out steps required to implement the social robot) | Inclusion Criteria: Include statements related to belief in their own capabilities (confidence in their ability) to execute courses of action to achieve implementation goals |
| Individual stage of change | Characterisation of the phase an individual is in, as he or she progresses toward skilled, enthusiastic, and sustained use of the social robot | Inclusion Criteria: Include statements relating to the characterisation of the phase an individual is in, as he or she progresses toward skilled, enthusiastic, and sustained use of the social robot |
| Individual identification with the organisation | A broad construct related to how individuals perceive their setting/organisation, and their relationship & degree of commitment with the implementing setting/organisation | Inclusion Criteria: Include statements relating to how individuals perceive their setting/organisation, and their relationship & degree of commitment with the implementing setting/organisation |
| Other personal attributes | Definition: A broad construct to include other personal traits such as tolerance of ambiguity, intellectual ability, motivation, values, competence, capacity, and learning style. | Inclusion Criteria: Include statements that include other personal traits such as tolerance of ambiguity, intellectual ability, motivation, values, competence, capacity, and learning style. |

1. **Process**

| **CFIR Constructs** | **Description** | **Inclusion and/or Exclusion Criteria** |
| --- | --- | --- |
| Planning | The degree to which a scheme or sequence of tasks for implementing the social robot are developed in advance, and the quality of those schemes or tasks. | Inclusion Criteria: Include evidence of pre-implementation diagnostic assessments and planning, as well as refinements to the plan. |
| Engaging | Attracting and involving appropriate individuals in the implementation and use of the intervention through a combined strategy of social marketing, education, role modelling, training, and other similar activities. | Inclusion Criteria: Include statements related to engagement strategies and outcomes (i.e. if and how stakeholders became engaged with the social robot and what their role is in implementation).  Exclusion Criteria: Exclude statements that are captured in the sub-codes below. |
| (i) Opinion leaders | Individuals in the care setting/ organisation who have formal or informal influence on the attitudes and beliefs of their colleagues with respect to implementing the social robot | Inclusion Criteria: Include statements related to engagement strategies and outcomes (e.g. how the opinion leader became engaged with the social robot and what their role is in implementation).  Double code statements to the CFIR sub-construct **“Leadership Engagement”** (under the ‘Inner Setting’ domain) if the formally appointed internal implementation leader is also the organisational leader |
| i) formally appointed internal implementation leaders | Individuals from within the organization who have been formally appointed with responsibility for implementing the social robot as coordinator, project manager, team leader, or other similar role. | Inclusion Criteria: Include statements related to engagement strategies and outcomes (e.g. how the formally appointed internal implementation leader became engaged with the social robot and what their role is in implementation).  Double code statements to the CFIR sub-construct **“Leadership Engagement”** (under the ‘Inner Setting’ domain) if the formally appointed internal implementation leader is also the organisational leader |
| (iii) champions | Individuals who dedicate themselves to supporting, marketing, and ‘driving through’ an implementation, overcoming indifference or resistance that a social robot may provoke in the setting | Inclusion Criteria: Include statements related to engagement strategies and outcomes (e.g. how the champion became engaged with the social robot implementation and what their role is in implementation).  Double code statements to the CFIR sub-construct **“Leadership Engagement”** (under the ‘Inner Setting’ domain) if the champion is also the organisational leader |
| (iv) external change agents | Individuals who are affiliated with an outside entity who formally influence or facilitate the implementation of social robot in a desirable direction. | External change agents may include researchers, health agency staff, health professionals and hired consultants external to the organisation  Inclusion Criteria: Include statements related to engagement strategies and outcomes (e.g. how the external change agent became engaged with the social robot and what their role is in implementation (e.g. how they supported implementation efforts).  Double code **“Access to Knowledge & Information”** where the external change agent influences access to knowledge and information. Double code **“Available Resources”** where it influences the ability to engage external change agents. |
| (v) key stakeholders | Individuals from within the organization with responsibility for implementing the social robot | Note: Internal key stakeholders may include care workers, managers etc.  Inclusion Criteria: Include statements related to engagement strategies and outcomes, e.g., how internal key stakeholders became engaged with the social robot and what their role was in implementation.  Exclusion Criteria: Exclude statements related to internal stakeholders who act as a champion for the social robot and code **“Champions”**. Exclude statements related to internal stakeholders who have been formally appointed as implementation leaders and code to **“Formally Appointed Internal Implementation Leader”.** |
| (v) innovation participants | Individuals served by the organization that participate in use of the social robot | Inclusion Criteria: Include statements related to engagement strategies and outcomes, e.g., how participants became engaged with the social robot.  Exclusion Criteria: Exclude statements demonstrating (lack of) awareness of the needs and resources of those served by the organization and whether or not that awareness influenced the implementation or adaptation of the social robot and code to **“Needs and Resources”** |
| Executing | Carrying out or accomplishing the implementation according to plan. | Inclusion Criteria: Include statements that demonstrate how implementation occurred with respect to the implementation plan.  (Note: Executing is coded very infrequently due to a lack of planning. However, some studies have used fidelity measures to assess executing, as an indication of the degree to which implementation was accomplished according to plan). |
| Reflecting & evaluating | Quantitative and qualitative feedback about the progress and quality of implementation accompanied with regular personal and team debriefing about progress and experience. | Inclusion Criteria: Include statements that refer to the implementation team’s (lack of) assessment of the progress toward and impact of implementation, as well as the interpretation of outcomes related to implementation.  Note: ‘Reflecting and Evaluating’ is part of the implementation process; it likely ends when implementation activities end. It does not require goals be explicitly articulated; it can focus on descriptions of the current state with real-time judgment, though there may be an implied goal (e.g. we need to implement the social robot(s)) when the implementation team discusses feedback in terms of adjustments needed to complete implementation.  Exclusion Criteria: Exclude statements related to the (lack of) alignment of social robot related goals with larger organizational goals, as well as feedback to staff regarding those goals and code to “Goals and Feedback” |
