# Supplementary material for: Barriers and facilitators to the implementation of social robots for older adults and people with dementia: a scoping review
Source: BMC Geriatr. 2021 Jun 9;21:351. doi: 10.1186/s12877-021-02277-9 (PMC8191065; doi:10.1186/s12877-021-02277-9)
Supplement: Supplementary file 4 — Additional file 4. [file 12877_2021_2277_MOESM4_ESM.docx]

**List of articles excluded after full-text screening, with reasons**

| **Article** | **Author, year** | **Reason for excluding** |
| --- | --- | --- |
| "Kampai, go docking pleasei" - Large Scale Field Test of a Social Robot in Users' Homes | Toth et al, 2019 | Conference abstract, no full-text available |
| "THIS ISN'T ME!" - The Role of Age-Related Self- and User Images for Robot Acceptance by Elders | Dudek et al, 2020 | Single or once-off use/testing of social robot |
| A Feasibility Study of a Social Robot Collecting Patient Reported Outcome Measurements from Older Adults | Boumans et al, 2020 | Single or once-off use/testing of social robot |
| A multimodal robot game for seniors | Hansen et al, 2017 | Single or once-off use/testing of social robot |
| A socially assistive robot to support physical training of older people - An end user acceptance study | Werner & Krainer, 2013 | Conference abstract, no full-text available |
| A Telemedicine Robot System for Assisted and Independent Living | Koceska et al, 2019 | Single or once-off use/testing of social robot |
| Acceptability of a teleoperated android by senior citizens in Danish society: A case study on the application of an embodied communication medium to home care | Yamazaki et al, 2014 | Single or once-off use/testing of social robot |
| Acceptance of a minimal design of a human infant for facilitating affective interaction with older adults - A case study toward interactive doll therapy | Sumioka et al, 2020 | Single or once-off use/testing of social robot |
| Acceptance of an animaloid robot as a starting point for cognitive stimulators supporting elders with cognitive impairments | Greco et al, 2009 | Single or once-off use/testing of social robot |
| Acceptance of Social Robots by Elder People: Does Psychosocial Functioning Matter? | Baisch et al, 2017 | Single or once-off use/testing of social robot |
| Acceptance of social-emotional robots (SER): Do elderly people like it better than caregivers? | Baisch et al, 2015 | Single or once-off use/testing of social robot |
| Adding a Context Will It Influence Human-Robot Interaction of People Living with Dementia | Hendrix et al, 2019 | No barriers or facilitators |
| Adventures of harvey - Use, acceptance of and relationship building with a social robot in a domestic environment | Klamer et al, 2011 | No barriers or facilitators |
| An evaluation of a telepresence robot: User testing among older adults with mobility impairment | Wu et al, 2017 | Single or once-off use/testing of social robot |
| Assessment of perceived attractiveness, usability, and societal impact of a multimodal Robotic Assistant for aging patients with memory impairments | Gerlowska et al, 2018 | Single or once-off use/testing of social robot |
| Assistive robot enabled service architecture to support home-based dementia care | Khosla et al, 2014 | No barriers or facilitators |
| Assistive robots for socialization in elderly people: results pertaining to the needs of the users | D’Onofrio et al, 2019 | No social robot intervention |
| Attitudinal Change in Elderly Citizens Toward Social Robots: The Role of Personality Traits and Beliefs About Robot Functionality | Damholdt et al, 2015 | Single or once-off use/testing of social robot |
| Caregiver and social assistant robot for rehabilitation and coaching for the elderly | Perez et al, 2015 | Single or once-off use/testing of social robot |
| Carer’s perception on social assistive technology acceptance and adoption: moderating effects of perceived risks | Khaksar et al, 2019 | No barriers or facilitators |
| Changes in technology acceptance among older people with dementia the role of social robot engagement | Chen et al, 2020 | No barriers or facilitators |
| Communication Robots for Elderly People and Their Families to Support Their Daily Lives - Case Study of Two Families Living with the Communicaton Robot | Inoue et al, 2015 | Conference abstract, no full-text available |
| Companion robots in the healthcare sector | Klein, 2015 | Conference abstract, no full-text available |
| Cost effectiveness of using Paro, a therapeutic robotic seal, to reduce agitation and medication use in dementia | Mervin et al, 2018 | No barriers or facilitators |
| Deploying new technology in residential aged care: Staff members' perspectives | Cavenett et al, 2018 | No social robot intervention |
| Design and development of an interactive service robot as a conversational companion for elderly people | Manuhara et al, 2018 | Not used for older adults and/or people with dementia |
| Design of an Affordable Socially Assistive Robot for Remote Health and Function Monitoring and Prognostication | Johnson et al, 2019 | No social robot intervention |
| Design of the companion robot interaction for supporting major tasks of the elderly | Lee & Yoo, 2017 | Single or once-off use/testing of social robot |
| Design, implementation and field tests of a socially assistive robot for the elderly: HealthBot version 2 | Jayawardena et al, 2012 | No barriers or facilitators |
| Effects of a robot intervention on visuospatial hemineglect in postacute stroke patients - a randomized controlled trial | Karner et al, 2019 | No barriers or facilitators |
| Effects of robot-assisted activity for elderly people and nurses at a day service center | Wada et al, 2004 | No barriers or facilitators |
| Ethical Issues Raised by the Introduction of Artificial Companions to Older Adults with Cognitive Impairment - A Call for Interdisciplinary Collaborations | Portacolone et al, 2020 | No social robot intervention |
| Evaluating human-robot interaction using a robot exercise instructor at a senior living community | Lewis et al, 2016 | Single or once-off use/testing of social robot |
| Evaluating older adults' interaction with a mobile assistive robot | Mucchiani et al, 2017 | No barriers or facilitators |
| Evaluation of an Assistive Telepresence Robot for Elderly Healthcare | Koceski et al, 2016 | Single or once-off use/testing of social robot |
| Evaluation of human robot interaction factors of a socially assistive robot together with older people | Wener et al, 2012 | Single or once-off use/testing of social robot |
| Feasibility and effect of a therapeutic robot PARO on moods and social interaction in older adults with declining cognitive function | Yu et al, 2014 | Conference abstract, no full-text available |
| Humanoid Robot as a Companion for the Senior Citizens | Joglekar & Kulkarni, 2018 | No social robot intervention |
| Humanoid robots: Advantages of social robots in the assistance of elders | Rea & Sciutti, 2019 | Abstract, no full-text available |
| Implementation effectiveness of psychosocial and environmental care practices in assisted living | Miller et al, 2020 | Used other technology |
| Influence of social presence on acceptance of an assistive social robot and screen agent by elderly users | Heerink et al, 2009 | Single or once-off use/testing of social robot |
| Investigation of practical use of humanoid robots in elderly care centres | Shen et al, 2016 | No barriers or facilitators |
| Management of acute pain in dementia: a feasibility study of a robot-assisted intervention | Demange et al, 2019 | Single or once-off use/testing of social robot |
| MARIO project: Validation in the hospital setting | Donofrio et al, 2018 | No barriers or facilitators |
| Measuring the impact of age, gender and dementia on communication-robot interventions in residential care homes | Obayashi et al, 2020 | No barriers or facilitators |
| Measuring the influence of social abilities on acceptance of an interface robot and a screen agent by elderly users | Heerink et al, 2009 | Single or once-off use/testing of social robot |
| Meeting requirements of older users? Robot prototype trials in a home-like environment | Kortner et al, 2014 | Single or once-off use/testing of social robot |
| MobiKa - Low-Cost Mobile Robot for Human-Robot Interaction | Graf et al, 2019 | Single or once-off use/testing of social robot |
| New efficiency: Introducing social assistive robots in social eldercare organizations | Hasenauer et al, 2019 | No social robot intervention |
| Performance of daily activities by older adults with dementia: The role of an assistive robot | Begum et al, 2013 | Single or once-off use/testing of social robot |
| Playing a memory game with a socially assistive robot: A case study at a long-term care facility | Louie et al, 2012 | No barriers or facilitators |
| Preliminary experiments on the acceptability of animaloid companion robots by older people with early dementia | Odetti et al, 2007 | Single or once-off use/testing of social robot |
| Psychological implications of domestic assistive technology for the elderly | Cesta et al, 2007 | No social robot intervention |
| Refusal and acceptance of Paro-based therapy: A pilot study in patients with major neurocognitive disorders | Demange et al, 2017 | Abstract, no full-text available |
| Relating conversational expressiveness to social presence and acceptance of an assistive social robot | Heerink et al, 2010 | Single or once-off use/testing of social robot |
| Reliability and acceptability of using a social robot to carry out cognitive tests for community-dwelling older adults | Takeda et al, 2019 | No barriers or facilitators |
| ROBIN, a Telepresence Robot to Support Older Users Monitoring and Social Inclusion: Development and Evaluation | Cortellessa et al, 2018 | Single or once-off use/testing of social robot |
| Robot Exercise Instructor: A Socially Assistive Robot System to Monitor and Encourage Physical Exercise for the Elderly | Fasola et al, 2010 | No barriers or facilitators |
| Robot services for elderly with cognitive impairment: testing usability of graphical user interfaces | Granata et al, 2013 | Single or once-off use/testing of social robot |
| Robotic Companions for Older People: A Case Study in the Wild | Doering et al, 2015 | Single or once-off use/testing of social robot |
| Robotic System for Physical Training of Older Adults | Avioz-Sarig et al, 2020 | Single or once-off use/testing of social robot |
| Social acceptance of a teleoperated android: Field study on elderly's engagement with an embodied communication medium in Denmark | Yamazaki et al, 2012 | Single or once-off use/testing of social robot |
| Social and empathic behaviours: novel interfaces and interaction modalities | Marti et al, 2015 | No social robot intervention |
| Social media agency robot for elderly people | Kobayashi et al, 2018 | No barriers or facilitators |
| Socializing robots: constructing robotic sociality in the design and use of the assistive robot PARO | Sabanovic & Chang, 2016 | No social robot intervention |
| Socially assistive robot enabled personalised care for people with dementia in Australian private homes | Khosla et al, 2016 | No barriers or facilitators |
| Socially Assistive Robots in Service Innovation Context to Improve Aged-Care Quality A Grounded Theory Approach | Khaksar et al, 2015 | No social robot intervention |
| Suitability of healthcare robots for a dementia unit and suggested improvements | Robinson et al, 2013 | Single or once-off use/testing of social robot |
| Talking over the robot - A field study of strained collaboration in a dementia-prevention robot class | Jeon et al, 2020 | No barriers or facilitators |
| Teach Me-Show Me'-End-User Personalization of a Smart Home and Companion Robot | Saunders et al, 2016 | Single or once-off use/testing of social robot |
| The Attitude of Elderly and Young Adults Towards a Humanoid Robot as a Facilitator for Social Interaction | Sinnema & Alimardani, 2019 | Single or once-off use/testing of social robot |
| The CARESSES study protocol: Testing and evaluating culturally competent socially assistive robots among older adults residing in long term care homes through a controlled experimental trial | Papadopoulous et al, 2020 | No social robot intervention |
| THE EFFECTIVENESS OF A NON-PHARMACOLOGICAL INTERACTIVE INTERVENTION, USING THE NAO ROBOT, FOR DEMENTIA-RELATED APATHY | Demange et al, 2019 | Others) Conference abstract with no full-text |
| The impact of a companion robot in combatting loneliness in people with dementia living in residential care. The mario project | Casey et al, 2018 | Others) Conference abstract with no full-text |
| The impact of serious games with humanoid robots on mild cognitive impairment older adults | Manca et al, 2020 | No barriers or facilitators |
| The influence of a social robots persona on how it is perceived and accepted by elderly users | Bartl et al, 2016 | Single or once-off use/testing of social robot |
| The multi-modal interface of Robot-Era multi-robot services tailored for the elderly | Di Nuovo et al, 2018 | Single or once-off use/testing of social robot |
| Touch or talk?: Comparing social robots and tablet pcs for an elderly assistant recommender system | Hammer et al, 2017 | Single or once-off use/testing of social robot |
| Towards a robot for supporting older people to stay longer independent at home | Vincze et al, 2015 | Single or once-off use/testing of social robot |
| Towards Social Robots that Support Exercise Therapies for Persons with Dementia | Cruz-Sandoval et al, 2018 | No barriers or facilitators |
| Understanding robots' potential to facilitate piano cognitive training in older adults with mild cognitive impairment | Mois et al, 2020 | No barriers or facilitators |
| Understanding the Potential of PARO for Healthy Older Adults | McGlynn et al, 2017 | Single or once-off use/testing of social robot |
| Using Socially Assistive Human-Robot Interaction to Motivate Physical Exercise for Older Adults | Fasola & Mataric, 2015 | Single or once-off use/testing of social robot |
| Using telepresence for social connection: views of older people with dementia, families, and health professionals from a mixed methods pilot study | Moyle et al, 2019 | Single or once-off use/testing of social robot |
| Will Older Adults Accept a Humanoid Robot as a Walking Partner | Karunarathne et al, 2018 | Single or once-off use/testing of social robot |
